# Artificial Intelligence–Enhanced Wound Care to Improve Access, Efficacy, and Equity in Wound Care for Older Adults in Rural and Remote Regions of Canada

**DOI:** 10.2196/85644

**Published:** 2026-03-30

**Authors:** Courtney Genge, Basnama Ayaz, Shannon Freeman, Heba Tallah Mohammed, Robert D J Fraser, Ibukun-Oluwa Omolade Abejirinde, Deirdre O’Sullivan-Drombolis, Rebecca Brookham

**Affiliations:** 1 Aging in Place Challenge Program, National Research Council Canada, 1200 Montréal Rd, Gloucester, Ottawa, ON, K1A 0R6, Canada, 1 2898861656; 2 Dalla Lana School of Public Health, University of Toronto, Toronto, ON, Canada; 3 Swift Medical, Toronto, ON, Canada; 4 School of Nursing, University of Northern British Columbia, Prince George, BC, Canada; 5 Centre For Technology Adoption for Aging in the North, Prince George, BC, Canada; 6 Arthur Labatt School of Nursing, Western University, London, ON, Canada; 7 Institute for Better Health, Trillium Health Partners, Mississauga, ON, Canada; 8 Giishkaandago'Ikwe Health Services, Fort Frances, ON, Canada; 9 Brightshores Health System, Owen Sound, ON, Canada

**Keywords:** wound care, AI, equity, artificial intelligence

## Abstract

Wound care is an increasing global challenge, with older adults among those most affected. As populations age, the demand for effective and efficient wound care increases. Over the years, various wound assessment and care techniques have been developed, including digital wound care technology (DWCT), which uses innovative artificial intelligence (AI). Many older adults, especially those living in rural and remote areas, face significant barriers in obtaining timely and effective wound care, leading to poorer health outcomes and increased health care costs related to wound care. These challenges underscore the urgent need to implement wound care models that equitably improve access to care and enhance clinical outcomes, particularly for older adults, to promote healthy aging and age-in-place. Based on evidence from the literature and the initial implementation of a DWCT in 2 community health systems in Ontario, this viewpoint paper encourages clinicians and health care leaders to embrace and expand the implementation of an AI-driven DWCT to address inequities in access to high-quality, timely care. The experiences from these implementations indicate that the use of AI can support clinical decision-making and extend access to care for individuals in rural and remote communities in Canada. By leveraging DWCT powered by AI, health care providers can enhance the accuracy and consistency of wound assessments, improve communication, streamline care processes, and more effectively allocate resources, ultimately aiming to reduce disparities in wound care outcomes.

## Introduction 

Wound care represents a growing challenge worldwide, particularly impacting older adults [1]. As populations age, the demand for effective and efficient wound care increases, driven by the growing number of older adults with multiple chronic conditions that predispose them to complex wounds and longer healing times. Many older adults, especially those living in rural and remote areas, face significant barriers in obtaining timely and effective wound care, leading to poorer health outcomes and increased health care costs [2]. These challenges underscore the urgent need to implement wound care models, which equitably improve access to care and enhance clinical outcomes. Traditionally, wound assessment is conducted visually to evaluate anatomical, physiological, and mechanical domains [3] or with subjective methods such as the Pressure Ulcer scale for Healing (PUSH) and Pressure Sore Status Tool (PAST) scales; these methods are widely used, can lack accuracy and consistency, leading to variable wound evaluation reports when administered by different clinicians [4].

With advances in science and technology, a range of instruments has been developed to address the growing demand for wound care. Among these, noninvasive imaging techniques have received particular attention for their potential to support wound assessment and monitoring. A systematic review of 20 studies done in 2015 summarized available approaches, including 2D stereophotogrammetry and 3D imaging methods. The authors concluded that 2D imaging is a practical wound measurement technique, offering ease of use and reliable assessments of wound size and healing progress; however, they noted that its inability to capture wound depth limits its value for care planning [5]. Building on this, a 2022 scoping review of 156 global studies examined the development, evaluation, and implementation of digital health technologies (eg, imaging and measurement) for wound care [6]. This review highlighted growing adoption of digital tools, citing evidence of their acceptability, feasibility, effectiveness, and clinical impact. At the same time, it identified important challenges, including limited evidence in hard-to-reach populations (eg, those in remote regions), low uptake linked to noninclusive design processes, and uncertainty around cost-effectiveness. It is essential to note that this review was not specifically tailored to older adults, a population at elevated risk of wound development and delayed healing due to comorbid conditions, and who also experience disproportionate barriers to care arising from age-related, geographic, and socioeconomic factors.

This study is a forward-looking perspective that highlights the current context of wound care in Canada and explores the potential for artificial intelligence (AI) driven digital wound care technology (DWCT) to improve access, efficiency, and equity. The authorship of this study reflects the multiple perspectives shaping this work, including 2 community health systems implementing a DWCT, the evaluation team supporting that implementation, academic and government partners advancing digital health innovation, and representatives from the company developing the technology. By bringing together these complementary experiences, we aim to provide a salient commentary on the merits of AI-augmented DWCT and its potential to advance equity, access, and efficiency in wound care in Canada.

The initial implementation of a DWCT in 2 rural and/or remote community health care systems, presented here as case studies, illustrates the contextual challenges faced by health care providers and patients while also offering practical insights into the technology’s application and benefits. Discussion of anticipated challenges in implementing AI technologies in wound care provides a roadmap for future research directions, including identification of potential facilitators and barriers to adoption and uptake of the DWCT in the 2 networked community health systems. The potential for AI in transforming health care delivery is vast, particularly in the field of wound care, and can play an important role in ensuring that all patients receive high-quality, equitable care across diverse geographies.   

## Wound Care in Canada

The Canadian population is experiencing a significant demographic shift, with older adults (65 years and older) rapidly becoming the fastest-growing demographic, and by 2051, they could represent 25% of the population [7]. Aging is associated with the accumulation of cellular and molecular damage within the body, which can manifest as frailty and/or chronic health conditions [8], which increases vulnerability to complex wounds, meaning the number of older adults needing wound care is anticipated to increase substantially in the future [9]. Although the exact prevalence of wounds requiring care in Canada is unclear, estimates suggest that up to CAD $11.1 billion (US $8.18 billion) are spent annually on wound care [9]. Approximately 1 in 500 Canadians develops nonhealing wounds [10], which significantly impacts the health care system [11]. In Ontario alone, 30%‐50% of health care delivery involves patients with wounds, predominantly affecting older adults who often heal at home [11].

In 2019, older adults constituted 17% of Canada’s population but accounted for 47% of total health care costs [10]. Rural and remote areas of Canada are aging more rapidly than their urban counterparts [12]; yet, they often have fewer health resources, especially health human resources (HHR) [2]. This lack of access to timely treatment for complex wounds leads to improper treatment of up to 25% of Canadian patients [13]. Chronic wounds impose significant physical and emotional burdens, decreasing patients’ quality of life and increasing health care costs [14,15]. Without regular, high-quality care, these wounds can lead to serious complications such as infections, hospitalization, amputation, and even death [1].  

 The complexity of wound care is exacerbated by limited opportunities for comprehensive wound care education for health care providers, who typically receive fewer than 10 hours of formal instruction [16]. During training, health care providers are taught to provide quality care and maintain optimal functioning, including the treatment of health conditions such as wounds, through supportive, preventive, therapeutic, palliative, and rehabilitative care. However, these professionals need additional training to fulfill the specific needs of care and their respective professional scope of practice. For example, the College of Ontario of Nurses specified the scope of practice for registered nurses (RNs), registered practical nurses (RPNs), and nurse practitioners (NPs), which includes 5 regulated acts, including performing a prescribed procedure below the dermis or a mucous membrane [17]. While this authorized act consists of wound assessment and wound care, which are at the entry level, nurses with advanced education, such as NPs, have an extended scope of practice and are authorized to diagnose, prescribe medications, and other treatments for clients [17]. Similarly, British Columbia has specified the pacified authorities at entry level and advanced level, which is presented in the Wound Care Canada by Freeman et al [18]. In addition, the British Columbia Provincial Nursing Skin & Wound Committee, in collaboration with the wound care clinicians from across all health authorities, has devised the wound assessment and treatment flow sheet: documentation guide (version 2, 2018) that specifies nurses’ roles with regard to wound assessment and documentation for varying types of wounds [19].

Traditional methods for tracking wound healing progress, such as using a paper ruler and cotton swab for wound assessment, result in high clinical variability and a 44% error rate in infection identification [20]. Furthermore, health care education and wound care tools have traditionally been developed with lighter skin tones in mind, leading to inaccurate assessment for patients with darker skin [21]. Additionally, rural communities in Canada are diverse, not only in terms of geographic characteristics such as population size, density, or distance from urban centers, but also in social aspects, including social representation and resource availability [18,22,23]. There is growing concern regarding the shortage of health care providers in remote and rural regions, further intensifying the issue of care access for older adults. The Canadian Institute for Health Information reports on the declining trends of health professionals in rural or remote areas, including for nurses, NPs, and pharmacists over a decade, and stagnant growth for rural family physicians [2]. Reducing health disparities, particularly in rural and remote areas with finite resources, requires innovative solutions, including consultation models and enhanced access to best practice care [24]. These dual drivers, population aging and uneven resource distribution, underscore the urgent need for effective health care solutions that:    

1. Enable individuals to age in place while living with aging-related health conditions, including chronic and complex wounds. 
2. Support the provision of best-practice clinical care in areas where specialized health care human resources are limited.
3. Reduce the challenges associated with human error and biases.   

To meet these needs, innovative technologies such as AI offer promising solutions that can enhance efficiency, access, and quality in health care delivery.

## AI-Enhanced Wound Care as a Solution  

AI has the potential to transform medical practices and service delivery in health care, including enhancing productivity, improving patients’ flow, experience, care, and quality of life [25] and enhancing providers’ experiences and safety [25,26]. The positive impacts of AI in clinical care include enhanced accuracy in diagnostics, improved patient engagement, and increased treatment support [25].  AI has been leveraged in wound care, providing more efficient and effective solutions for acute and chronic wounds [27]. AI-driven algorithms have proven effective in measuring wound dimensions and identifying prognostic features, such as tissue composition, granulation, slough, eschar, and exudate. These elements are crucial for assessing wound burden and predicting the healing trajectory [28]. 

## Our Implementation Experience: Project and Team

To illustrate how AI-augmented wound care can enhance equity, access, and efficiency, we draw on our collective experiences implementing a DWCT in 2 networked Canadian community health systems. The following section describes the technology itself, the project sites, and the multidisciplinary team involved in its adoption, highlighting both practical considerations and lessons learned in real-world settings.

## The Digital Wound Care Technology 

The DWCT under implementation, Swift Skin and Wound (Swift), is developed by Swift Medical Inc. and is deployed in more than 5200 health care facilities internationally, spanning the continuum of care. Swift Skin and Wound is a software-based, noninvasive digital wound assessment application installed on smart devices. The software captures clear imaging enhanced by HealX (Swift Medical Inc), a Health Canada and US Food and Drug Administration–registered fiducial marker for real-time calibration of wound images on smart devices, using AI-driven algorithms that auto-trace wound edges, precisely measure wound surface area, and facilitate comprehensive documentation [29]. These features offer advanced analytical and tracking insights to clinicians, empowering them with data-driven assessments to support wound care management [28,29]. This DWCT also enhances communication among the interdisciplinary care team through the centralized dashboard portal, which provides a comprehensive, real-time view to support coordinated care management. The dashboard enables the care team to review patient information, track progress, and provide recommendations, facilitating coordination of care. 

**Figure 1.**
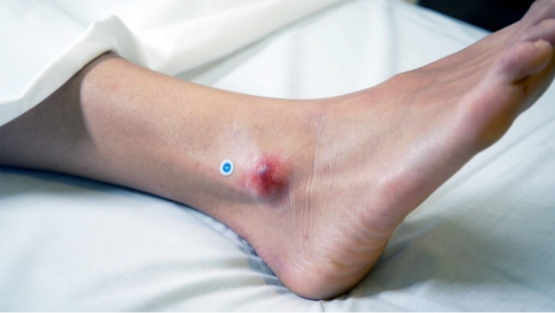
A sample wound on the ankle area, showing a clinical presentation where objective and consistent wound assessment, supported by an artificial intelligence–driven tool for measurement and tissue classification, can enhance effective monitoring, especially in settings with limited access to specialized expertise.

Recently, Swift Skin and Wound added additional features: AutoDepth, SmartTissue, and HealingIndex. AutoDepth automatically calculates the visible depth of wounds, measures the wound, and accurately identifies and records its deepest point on a smart device. SmartTissue detects and quantifies tissue types including epithelial, granulation, slough, and eschar within the wound bed, irrespective of skin tone, using deep learning and vision architectures designed to run locally on a wide range of smart devices [30]. HealingIndex uses deep learning and machine learning algorithms to analyze a range of wound characteristics, including wound size, tissue composition, wound type, location, and wound exudates’ type and amount to predict healing trajectories of wounds [28]. By using these sophisticated AI-driven algorithms, providers can achieve rapid and precise assessments of wounds and their characteristics and use predictive modeling to project healing trajectories and inform clinical decision-making for care plans (Figure 1). 

Evidence from implementations, mostly in US health systems, indicates that integrating Swift’s DWCT into wound care programs can improve clinical outcomes [31,32], enhance operational efficiency [29,32], reduce costs [31-33], and increase clinician satisfaction [34].

While other digital wound care technologies exist, this viewpoint focuses on Swift’s DWCT because the authorship team is uniquely positioned as active implementation partners in Canadian health systems. We draw on the published literature to provide context and evidence, but we triangulate these insights with our direct, real-world experience to highlight equity, access, and efficiency considerations that emerge in practice, providing insights that would be difficult to capture from literature alone.

## Project Description  

This project uses an implementation research approach to evaluate the adoption of the DWCT in 2 networked Canadian health systems, aiming to identify facilitators, barriers, and lessons for broader AI-driven wound care implementation.    

### Implementation Site 1: Giishkaandago'Ikwe Health Services 

Giishkaandago’Ikwe Health Services (formerly Fort Frances Tribal Area Health Services) is an Indigenous-led health organization in Northwestern Ontario. The accredited organization provides holistic health services to the 10 Anishinaabe Communities of Southern Treaty 3. The Home and Community Care Program offers essential services in the home and community setting, including advanced wound care through a nurse-led DWCT tool.

Giishkaandago’Ikwe initiated implementation of the DWCT in February 2022 as a quality improvement initiative. This site has particular expertise in leveraging technology to provide care across remote geographic regions. Given the high amputation rates in the region, the home and community care team implemented the DWCT to strengthen decision-making for wound management through efficient documentation and communication, with the goal of enhancing healing rates and reducing amputations.

The interdisciplinary wound care team includes RNs, RPNs, including 2 Skin Wellness associate nurse (SWAN)–trained RPNs, personal support workers (PSWs), and a physiotherapist with advanced wound care training (Masters of Clinical Science in wound healing). In total, the team includes 14 wound care clinicians, supported by a wound care champion and a home and community care coordinator. Foot care nurses conduct wound assessments and communicate findings to the home care team, while other community health nurses contribute through public health programming and wound care support.

Team members complete an 8-hour online wound care training upon hire if they do not already have advanced wound care education. In addition, the team participates in an annual, full-day, hands-on internal wound care workshop to reinforce clinical skills. Weekly SWIFT rounds provide structured opportunities for case review, collaborative problem-solving, and ongoing knowledge exchange.

Implementation of the DWCT was completed over 1 month, supported by training and mentorship from both SWIFT and the site’s internal champion (the home and community care coordinator). Initial training consisted of a 1-hour virtual session, followed by ongoing support throughout the implementation period. Due to the team’s small size, strong collaboration, and existing wound care expertise, adoption of the technology was rapid and well integrated into practice. The AutoDepth feature has been actively used in patient care, and team members completed SmartTissue training in January 2025.

### Implementation Site 2: Brightshores Health System (Brightshores)

Brightshores Health System is a comprehensive health care network serving the rural Gray and Bruce counties in Ontario, Canada. It operates 6 hospitals (Owen Sound, Meaford, Markdale, Southampton, Wiarton, and Lion’s Head), each with 24/7 emergency departments. Brightshores services encompass medical and surgical services, including complex surgeries, total joint replacements, cancer treatments, advanced diagnostic imaging such as magnetic resonance imaging and computed tomography scans, and wound care. Across the system, over 400,000 visits per year are reported, and approximately 2200 staff members with over 250 dedicated physicians deliver patient care across the region, including wound care.

Implementation of the DWCT began in 2024, initially within the regional diabetic foot ulcer clinic. Within months, 35 clinical staff were trained as technology champions, and by October 2024, the DWCT was rolled out to multiple departments across all 6 hospitals. Initial training was performed in-person (2.5 h) with an asynchronous online portion (1.5 h) facilitated by the research team and a nurse specialized in wound, ostomy, and continence (NSWOC), with a focus on use of the AI technology, clinical data entry education, and workflow processes. Users included RNs, RPNs, clinical coordinators, NPs, nurse clinicians, and PSWs. Within 6 months, it was noted that the turnover of staff necessitated continuous training opportunities. An online training module has been developed specifically for the Brightshores sites, which will be more accessible and enable quicker onboarding for staff across multiple sites. Deployment of SmartTissue began in December 2024 and continues across sites.

## Discussion

### AI-Powered Digital Wound Care Could Improve Access, Enhance Efficiency, and Address Inequities  

#### Overview

Wound care is a critical component of comprehensive home health services. The prevalence of older adults as recipients of home care is notable, with a substantial proportion necessitating wound care due to conditions such as pressure injuries, postsurgical recovery, and complications arising from venous ulcers. Older adults prefer to receive wound care in community settings [35], citing pain, transportation barriers, and risk of exposure to infection in the clinic as primary drivers of preference [36]. Patients and family caregivers frequently report feeling inadequately prepared to participate effectively and confidently in wound care [37]. The presence of nonhealing wounds and associated complications can profoundly affect quality of life, increasing risk of infections and hospitalization [38].    

#### Access  

In the Canadian context, the potential for an AI-enhanced DWCT to increase access to best practice care is particularly significant.  The “Caring for Canadians” study highlights a significant gap between the supply and demand for HHR in Canada, a gap that is already evident and expected to widen over the next decade, with rural and remote communities feeling the impacts most acutely [39]. Specialist HHR, such as NSWOCs or additional training in wound care, is in high demand. However, recruiting and retaining specialized health care workers is challenging, which has led to the adoption of innovative, technology-enhanced care models to optimize resource use [2,24].

In Canada, geographic location is a primary factor determining access to care, with isolated and remote communities experiencing the highest levels of deprivation [40]. These communities are frequently located in northern regions of the provinces and territories and are often home to Indigenous groups [40]. First Nations, Métis, and Inuit people, along with those of African, Caribbean, Southeast Asian, and Latin descent, have higher prevalence and incidence of diabetes and higher rates of lower extremity amputation because of diabetic foot ulcers and peripheral arterial disease [41]. This is particularly evident in Indigenous populations, where the rate of major amputations due to diabetic wounds is up to 49 times higher than in non-Indigenous populations [42]. This may be attributable to poor access to health care, barriers to clear communication, cultural differences, and discrimination [43]. Within this context, the AI-augmented DWCT offers an opportunity to increase access to best practice care by leveraging available resources and supporting available staff to work at the frontiers of their scope of practice (Textbox 1). 

Textbox 1. **Case study: Giishkaandago’Ikwe Health Services.**

The team at Giishkaandago’Ikwe Health Services uses digital wound care technology (DWCT) for all aspects of wound assessments. Leveraging this technology has strengthened communication internally and externally with care partners. Enhanced communication is central to improved outcomes, illustrated here through a client story.

A client was referred by the Emergency Department after seeking care for painful, reddened foot wounds that had worsened following several weeks of self-treatment. The team initiated a comprehensive wound assessment completed by a personal support worker (PSW) using the SWIFT app. PSWs are unregulated care providers who assist individuals with activities of daily living (ADLs) and instrumental activities of daily living (IADLs).

A holistic treatment plan was then created by the interdisciplinary team during SWIFT rounds. Giishkaandago’Ikwe Health Services mobilized available staff to provide care, ensuring they were supported to work to their full scope of practice. The client observed weekly progress through the app, which increased their engagement and adherence to the care plan. The team also used dashboard-generated reports to share wound progress with external health care providers.

Through this collaboration, the client’s care was escalated appropriately, enabling timely access to vascular surgery and chiropody for specialized offloading. The client received these services in a tertiary center and was able to return home promptly, with the Giishkaandago’Ikwe team providing ongoing follow-up.

Despite a late start to wound care and the need for surgical intervention, the client’s wounds fully healed within 5 months—an excellent outcome for a case at high risk of amputation. Importantly, the majority of the client’s care occurred within their home community, minimizing disruption to their family and caregiving responsibilities. The ability to coordinate and communicate effectively across providers using DWCT was key to avoiding prolonged hospitalization and ensuring continuity of care close to home.

In the Giishkaandago’Ikwe Health Services case study, the DWCT enabled a PSW who had direct contact with the patient to complete a wound care assessment even though this typically would be conducted by nursing staff. The DWCT tool enabled the team member who was geographically co-located to effectively capture the data needed for the wound care team to create a full care plan, without requiring the patient or the specialist providers to travel.  In such scenarios, AI-augmented DWCT can support an available staff member to work at the frontiers of the scope of practice while maintaining direct connections for clinical escalation and consultation when needed.   

 This is particularly relevant in the Canadian rural and remote context, where geography and inclement weather can act as a barrier to access. A 2020 review of barriers to equitable access identified the lack of year-round roads, extreme winter weather events, and the absence of rural and remote hospital staffing as key contributors to poor access to care in Indigenous communities [44]. That same review identified telehealth and telehealth-enabled consultation models as a mitigator of inequitable access. Furthermore, the review noted that telehealth enables community health workers and nurses to provide additional services in their local community and reduces the burden on patients to travel long distances to access care. This has important implications for equitable access, as community care providers typically have higher levels of trust and greater ability to provide culturally safe care [45].

The DWCT augments traditional “telehealth” in wound care by adding empirical data in the form of high-quality, time-lapsed photos in a “dashboard” that can be accessed by patients, caregivers, and the entire wound care team. This enables tracking of wound trajectory and gives contextual clinical data to geographically remote specialist providers such as NSWOCS, endocrinologists, and vascular surgeons.  Additionally, the dashboard supports team communication as it offers a shared point of reference to facilitate collaborative care planning.  In the context of an optimized scope of practice, this feature provides an additional layer of protection—ensuring specialized clinical oversight of geographically remote patients being cared for by practitioners working at the frontier of scope of practice.  Furthermore, this supports staff who may not have clinical training in wound care by providing avenues for consultation and escalation to complement their clinical judgment.  Ultimately, this benefits patients who receive care from a trusted provider in their community, while retaining the benefit of review and consultation from clinical experts.  

The AI-augmented features of the DWCT expand access to best practice care by (1) enabling patients in remote communities to access services without necessitating travel, (2) empowering clinicians practicing in rural and remote communities to work with an optimized scope of practice, and (3) supporting communication and ensuring all members of the care team have access to relevant clinical information. 

####  Efficiency  

 Optimization of clinical resources is required to ensure efficiency and maximize the number of patients who receive best practice care. The AI-enhanced DWCT tool can increase clinical efficiency by conducting rapid assessment of wounds and streamlining consultations and escalations [28]. A key component of effective wound care is assessment of wound size. Traditionally, this is done with paper rulers, and the wound size is calculated using a formula that multiplies length by width. However, this traditional method is ill-suited to irregularly shaped wounds, leading to overestimation of size in 36%‐74% of nonrectangular wounds [46-48]. Patients with darker skin tones, wounds characterized by diffuse edges, irregular shapes, necrotic tissue, or unhealthy surrounding tissue are more prone to having their wounds overestimated in size [46]. A more accurate calculation of wound size can be captured using a tracing method, where transparency film is placed over the wound and outlined, but this is reported to be time-consuming [49].  Accurate and objective measurement of wounds is essential as a change in wound size is a key predictor of wound healing trajectory [5]. Digital wound measurements powered by AI are emerging as a more efficient and accurate means to measure wounds [46,50-52] (Textbox 2).

Textbox 2. **Case Study: Brightshores Health System.**

Brightshores Health System is spread throughout 8600 square kilometers in Southwestern Ontario. Residents are among the oldest average age in the province, with the fastest-growing segment of the population aged between 65 and 84 years. The region has a significant population of patients with chronic disease and wounds, but Brightshores is limited to 1.4 Nurses Specialized in Wound, Ostomy, and Continence (NSWOC’s) for the 6 hospital sites.

This means that daily, multiple wounds can present at any one of Brightshores hospitals, and the reality of limited resources means limited and delayed response time. Furthermore, without access to specialists and with limited tools, frontline nursing staff struggle to accurately assess wounds. The DWCT has presented an opportunity for frontline staff to take photos of wounds, allowing for accurate and detailed assessments that can be digitally accessed by the NSWOC and all providers within the circle of care. Providing frontline staff at all 6 hospital sites with a tool to accurately assess wounds allowed for a reduction in reliance upon the NSWOC.

Within the first year, over 1000 km of NSWOC travel time has been saved, allowing them to devote more time to direct patient care. Since the implementation of this DWCT, initial trends show a promising decline in amputation rates and fewer visits of outpatient wound care patients attending the emergency departments due to wound complications, compared to the previous year. Patients are reporting that their assessments performed with the DWCT are more comfortable than traditional methods and are reporting that the use of DWCT has improved their wound care experience.

In one patient's story, the patient felt discouraged about the lack of visual improvement they were able to detect with their eyes. With the use of the DWCT and historical measurements, the patient was able to monitor progressive wound closure, aligning with compliance to treatment recommendations, and was happy to experience a full wound closure within a month of using Swift.

Brightshores' continued hope is that the use of this technology will empower nurses to gain confidence and become more involved in wound assessments, enabling prioritization of wound care, and that this technology will enable patients to see progression in healing over time.

This case study highlights the benefits of tools that enable the clinical care team to optimize their time and streamline processes for escalating concerns to specialized care resources. Use of the DWCT is faster and clinically equivalent in accuracy compared to manual methods of wound measurement and capture [29,53]. Enhanced DWCT reduces the overall time required for wound measurement and documentation and has been shown to result in an average time saving of 1.01‐2.39 minutes per wound assessment when compared to the traditional manual methods [29]. Given the known constraints on clinicians’ time, burden of documentation, and anticipated workforce shortages, these time savings represent an opportunity to increase both efficiency and access.  

AI tools can also help identify and escalate wounds that require review.  HealingIndex   is a new feature of the DWCT that uses machine learning algorithms to assess the photographs of the wounds that are submitted by clinicians. This tool analyzes the photo to identify wound characteristics associated with healing including wound size, tissue composition, and exudates, to predict the healing trajectories of the wound. The HealingIndex AI Model uses deep learning techniques to examine patient wound records, images, and characteristics, producing a score from the AI model that indicates the level of risk for delayed healing and potential deterioration [28].

 While traditional care pathways rely on clinicians identifying and escalating wounds that are deteriorating or at risk of deteriorating, the HealingIndex automates these processes using a predictive algorithm. This allows the wound care team to rapidly identify and prioritize wounds that require specialist input. This identification reduces the risk of deterioration and complications that necessitate hospitalization or amputation, reducing overall health care costs associated with wound complications [1,31,38]. Furthermore, the use of this AI tool can identify wounds at risk for slow healing, which may require an adapted wound management protocol. By reducing complications and adapting the treatment of slow-healing wounds, the AI tool can reduce the overall costs associated with wound care [31]. Early identification of wound deterioration is crucial as it allows timely intervention, preventing progression and potentially leading to significant savings for the health care system through avoidance of unnecessary costs [34]. 

The use of the predictive algorithm may also augment clinical decision-making by identifying wounds that are healing effectively and therefore may not need daily or thrice-weekly dressing changes. Reducing the frequency of dressing changes reduces supply costs and frees patient and provider time. Reducing the frequency of dressing changes to once a week, where clinically appropriate, can free up thousands of hours of nursing time over the course of a year [54]. Overall, the AI-powered DWCT can enhance clinical efficiency by automating escalation and review processes.  

Additionally, the DWCT platform provides opportunities for asynchronous collaboration. Specialists are able to review patient records, including high-resolution time-lapse photos, and provide recommendations without requiring geographic co-location or congruent timing. This enables the specialist wound care nursing team to optimize their time by reducing travel between sites, reducing administrative burden, and creating care plans that can be executed by staff located near the patient. This offers the benefits of not only efficiency but also equity, as access to best practice care and consultation is no longer limited to the fortunate few who can be reviewed by the specialist nurses in person. In rural and remote parts of Canada, which, as noted above, are struggling to recruit and retain skilled providers, these tools enable more patients to access specialist consultation and review without necessitating travel.  

AI-powered DWCT can support increased efficiency by (1) reducing time spent on manual assessment and measurement of wounds; (2) automating escalation by flagging deteriorating wounds for review, which enables prioritization of at-risk patients for intervention; and (3) enabling collaboration between frontline staff and specialist wound care resources without requiring geographic colocation or time alignment.  

#### Equity 

Universality and accessibility are embedded within Canada’s Health Act, and equity must be centered as a priority.  Inequities in the Canadian health system exist, especially related to disparities in health outcomes for rural and remote populations, and disproportionately affect First Nations, Inuit, and Métis communities who face burdens associated with travel and costs [44]. Rural health care policies are often guided by urban care models, which can exacerbate these inequities for rural communities [24].

Many rural, remote, and Indigenous communities experience inequitable access and poor outcomes, which may be attributable in part to resource shortages. Additionally, human biases, racism, colonial legacies, and historically unbalanced relationships between service users and providers also play a key role. Nyugen et al [44] explicitly state “Colonization and historical intergenerational traumas... have plagued the survivors and later generations with physical and mental trauma,” and “...many Health Care providers do not acknowledge the impact of colonization on the Indigenous community and disregard the social determinants of health as explanations for illness.” This, in turn, manifests as culturally inappropriate health services, which deter people from seeking health care. 

 In this context, the AI-enhanced DWCT can support more equitable care by enabling community care providers with existing, trusting relationships to provide culturally safe care.  This approach is aligned with mitigation strategies proposed by Indigenous health experts who highlight the importance of community partnerships and provision of culturally safe care [44,45]. 

Furthermore, patients with darker skin tones disproportionately experience poor wound outcomes, which may be attributable to both inequities in social determinants of health and limitations in provider ability to accurately identify clinically relevant changes in tissue in pigmented skin [55].  Johnson et al [55] note that erythema is an excellent example, as its presentation varies based on skin tone, and historically, medical education has failed to account for diverse skin tones in training and published literature [21]. Darker skin tone is associated with overestimation of wound size when using standard “width X length” measurements [46]. In comparison, the DWCT with AI-augmented AutoTrace, AutoDepth, and SmartTissue is able to provide accurate measurements and can enable color calibration to adjust for light conditions and skin pigmentation [46]. Thus, the use of the AI-enhanced DWTC can support more accurate measurements and potentially overcome some of the implicit biases that health care providers manifest. 

#### Next Steps 

While AI-enhanced tools offer immense promise to support better care efficiency and outcomes, there are several key considerations that must be addressed to ensure equitable and trustworthy implementation. One of the pressing concerns is the representation of diverse patient populations in AI systems. When datasets fail to capture diversity across factors such as skin tone, age, and other demographic variables, resulting models can produce biased or inaccurate outputs. These biases are particularly problematic in clinical contexts like wound care, where diagnostic accuracy is critical. Diverse groups, such as individuals with darker skin tones or older adults, are often underrepresented in training datasets, leading to biases in AI outcomes. This underrepresentation can result in inaccurate diagnoses or recommendations, which are harmful and counter to the goals of quality wound care. Human biases embedded in AI systems can become enshrined or even amplified if not intentionally designed with equity in mind [25]. Such biases arise when AI models are trained on datasets that do not adequately represent the diversity of the population, including those most likely to experience wounds, such as older adults [56]. Chu et al [56] emphasize the known challenges of ageism in AI, noting that models often overlook older adults due to insufficient data representation. To counteract these biases, it is essential to increase the diversity of AI training datasets and maintain robust evaluation protocols that ensure accuracy and interrater reliability across patient populations. These safeguards are crucial in mitigating the risks of bias and ensuring that AI systems are fair and inclusive. 

In addition to addressing bias, overcoming skepticism and implementation barriers is crucial for the successful adoption of AI-enhanced technologies, particularly among older adults. While the stereotype of older adults as technophobic is increasingly being disproven [57], structural barriers such as the cost of technology and access to reliable internet remain significant hurdles for many older adults in rural and remote communities [58]. Despite these challenges, patients are generally open to the use of AI in health care if appropriate regulatory and safety oversights are in place [59]. Pilot studies, such as those by Wang et al [52] and Raismam et al [60], show promising results regarding patient acceptance of AI for wound care [52,60] while Mohammed et al [34] highlight positive provider acceptance [34]. These studies indicate a growing receptiveness toward an AI-augmented DWCT, but continued exploration into unique barriers, especially those pertinent to the Canadian context, is necessary.  

The evaluation being conducted by this team will enhance understanding of implementing AI-enhanced DWCT in rural and remote communities in Canada. This evaluation will identify specific barriers and facilitators of adoption, ultimately leading to the creation of a framework that supports wider adoption and integration of technologies to support healthy aging. By addressing these issues, the path toward equitable, efficient, and widespread use of AI technologies in health care becomes clearer, fostering a more inclusive technological future. 

## Abbreviations

AI: artificial intelligence
DWCT: digital wound care technology
HHR: health human resource
NP: nurse practitioner
NSWOC: Nurses Specialized in Wound, Ostomy, and Continence
PAST: Pressure Sore Status Tool
PSW: personal support worker
PUSH: Pressure Ulcer Scale for Healing
RN: registered nurse
RPN: registered practical nurse
SWAN: Skin Wellness associate nurse
